# HIV and hepatitis C Virus in internally displaced people with and without injection drug use experience in the region of Shida Kartli, Georgia

**DOI:** 10.1186/s13104-024-06891-9

**Published:** 2024-10-20

**Authors:** Adrian Trovato, Maka Gogia, Ana Aslanikashvili, Tamuna Kasrashvili, Ganna Kovalenko, Anna Yakovleva, Britt Skaathun, Tetyana I. Vasylyeva

**Affiliations:** 1 Department of Population Health and Disease Prevention, University of California, Irvine, CA USA; 2 School of Public Health, San Diego State University, San Diego, CA USA; 3 National Center for Disease Control and Public Health, Tbilisi, Georgia; 4 Georgian Harm Reduction Network, Tbilisi, Georgia; 5 Division of Virology, Department of Pathology, University of Cambridge, Cambridge, UK; 6 Medical Sciences Division, University of Oxford, Oxford, UK; 7 Division of Infectious Diseases and Global Public Health, University of California San Diego, San Diego, CA USA

**Keywords:** Displacement, HIV, HCV, People who inject drugs, Georgia

## Abstract

**Objective:**

Internally displaced persons (IDPs) can have limited access to HIV and hepatitis C Virus (HCV) treatment and prevention. IDPs comprise > 7% of Georgian population but prevalence and levels of HIV and HCV knowledge in this population remain unknown. We tested 100 IDPs in Georgia for HIV and HCV, many of whom had drug injecting experience, and interviewed them about their migration experience, sexual and drug injecting practices, and HIV/HCV transmission knowledge.

**Results:**

The average age of participants was 37.5 years (range 18–63); 31% were women. Almost half (N = 48) of participants reported ever injecting drugs; 17% of those (N = 8) started injecting drugs within the last year. Anti-HCV and HIV prevalence was 11% and 0%, respectively. Fewer people without drug use experience compared to people who inject drugs correctly answered all questions on the HIV knowledge test (13% vs. 35%, *p* = 0.015) or knew where to get tested for HIV (67% vs 98%, *p* < 0.001). There was no difference in HCV knowledge between the two groups. HIV and HCV prevalence remains low among Georgian IDPs, but levels of HIV knowledge were much lower than levels of HCV knowledge.

**Supplementary Information:**

The online version contains supplementary material available at 10.1186/s13104-024-06891-9.

## Introduction

Internally displaced persons (IDPs) compose approximately 60% of forced migrants worldwide. Forced displacement impacts the distribution and spread of infectious diseases, including blood borne chronic diseases like HIV and hepatitis C Virus (HCV) [1]. Countries experiencing internal displacement often have limited resources, making infectious disease surveillance in IDPs challenging [2].

**Fig. 1:**
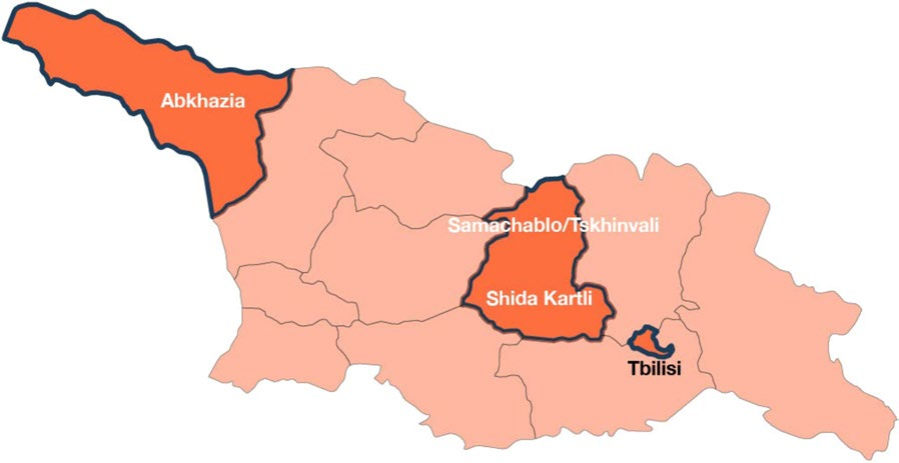
A map of Georgia, regions of origin and resettlement are marked in dark blue borders (adapted from Vemaps.com)

Since 1991 Georgia, an Eastern European country of 3.7 million, has experienced significant internal displacement; 286,000 people (7.7% of the total population) were IDPs in 2022 as a result of the occupation of Abkhazia since the early 1990s and South Ossetia since 2008 [Fig. 1] [3]. Displacement-associated stressors can cause depression/anxiety, and IDPs can resort to using drugs/alcohol to cope [4–7], which might put them at higher risk for HIV or HCV [8].

Georgia has been heavily affected by HCV since the 1990s [6]. In 2015, 7.7% of the adult population were estimated to be anti-HCV positive and 5.4%—HCV RNA/cAg positive [9]. In 2015, in response to high HCV prevalence, Georgia adopted a national HCV elimination program, aiming to reduce prevalence of HCV infection by 90% by 2020, which resulted in > 80% of those diagnosed (approximately 72,000 patients) being cured between 2015 – 2020 [10, 11]. In 2021, 6.8% of the adult population was estimated to be anti-HCV positive and 1.8% HCV RNA/cAg positive, indicating a 67% reduction in chronic HCV since 2015 [12]. As of 2020, HIV prevalence remains low (0.4%) in the general population in Georgia [13].

People who inject drugs (PWID) are often at higher risk for HIV and HCV; indeed, anti-HCV prevalence among PWID in Georgia was 58.1% in 2022 and 51.1% in 2015 (8, [14]. However, Georgia has lower HIV prevalence among PWID than many Eastern European/Central Asian countries (2.3% vs. 21.9% in Ukraine, 25.1% in Belarus, 18.5% in Latvia, 8.5% in Azerbaijan, and 48.3% in Estonia) [15]. Multiple interventions in PWID also resulted in high levels of HIV knowledge in PWID who are clients of the Georgian Harm Reduction Network (GHRN)(16).

Data regarding infectious diseases among IDPs is scarce and outdated if available; a 2004 study showed that prevalence and incidence of infectious and parasitic diseases among IDPs on average is 3 times higher than in the general population in Georgia[17, 18]. In 2006, IDPs represented a disproportionately high number of people living with HIV (PLWH); IDPs made up 5.5% of the Georgian population and 8.9% of PLWH [19]. To our knowledge, there is no previous data about HCV prevalence among Georgian IDPs. This study is the first to provide some data on HIV/HCV knowledge and awareness of testing and treatment, and current HIV/HCV prevalence among IDPs in Georgia.

## Methods

### Data collection

In June 2022, we conducted a cross sectional study at mobile ambulances near IDP villages in Shida Kartli region of Georgia. Shida Kartli hosts approximately 17,000 IDPs across 75 settlements (Collective Centres (CCs) [5]. (Fig. 1). The local non-governmental organization (NGO) “Step to Future” (STF) that provides harm reduction services in the area conducted recruitment. The GHRN compiled a list of all locations visited by the mobile ambulances, then randomly selected one at a time for recruitment. Participants were recruited through convenience and snowball sampling, non-probability sampling techniques used among vulnerable populations [20, 21]. Inclusion criteria were: (1) IDPs (2) age 18 + (3) residence in a CC in Shida Kartli. Each primary respondent received three uniquely identified coupons, then distributed the coupons to others. Participants could participate by redeeming their coupon at STF’s mobile ambulances. Our sample size of 100 was selected/saturated by available resources given that this was a pilot study; additionally, our calculations showed this sample size would be sufficient to detect differences as 0.05 statistical level with 80% power if HCV prevalence among IDPs was at least 2 times higher than the estimated 7.7% anti-HCV prevalence in the general population [9, 18, 19]. Participants were surveyed about their migration/displacement experience, number of sexual partners and sexual practices within the past 6 months, whether they had ever injected drugs, drug injecting practices within the past 30 days, knowledge of HIV/HCV transmission routes, knowledge of HIV/HCV treatment and prevention programs, and medical history regarding HIV/HCV. (See Supplemental Tables 1 and 2, and Supplementary File 1 for more detail about survey questions). Survey questions were adapted from bio-behavioural surveys conducted by GHRN in Georgia since 2002 [22] and from our previous work with IDPs in Ukraine [23, 24]. After interviews, participants were tested for HIV and HCV (rapid tests), and offered to receive harm reduction services, case management if applicable, and legal services from STF. Participants with a positive rapid HCV test result were promptly assisted to obtain HCV confirmatory diagnostics, and if confirmed positive, supported in initiating direct-acting antiviral (DAA) treatment.

### Measures

HIV and HCV transmission knowledge was measured by answering Correct/Incorrect to a set of questions (see Supplemental Table 1). As IDPs consistently report high levels of depression [25], the Patient Health Questionnaire depression scale (PHQ-9) (a tool with demonstrated high reliability (Cronbach’s α = 0.89)) was utilized [26].

### Statistical analysis

Socio-demographic and behavioural characteristics of IDPs were summarized with descriptive statistics. Continuous variables were presented as mean and range; categorical variables were presented as proportions. To compare IDPs with and without injection drug use (IDU) experience, we used a chi-square (χ^2^) test for categorical variables and analysis of Variance (ANOVA) test for continuous variable. *P*-values < 0.05 were considered as statistically significant. Depression score was measured as a continuous variable.

## Results

### Socio-demographic data

Socio-demographic and behavioural characteristics of the participants (N = 100) are presented in Table 1. The average age of participants was 37.5 (range 18–63); 31% were women; 59% had partial or complete secondary education. Nearly all participants were displaced from Abkhazia (94%); most of those following the escalation of the conflict in 2008 (83%). Eighty percent of participants settled in IDP shelters immediately after displacement. No participants tested positive for HIV; 11 participants received a positive rapid anti-HCV test in the study; of those, 3 participants tested HCV RNA/cAg positive when linked to care to receive DAA treatment.

**Table 1:** Socio-demographic characteristics of participants

		N/Mean	% (Range)
Socio-Demographic characteristics of participants			
IDP* Settlement	Poplars	21	21
	Karaleti	20	20
	Khurvaleti	30	30
	Scra	2	2
	Blacks	27	27
Gender	Female	31	31
	Male	69	69
Age		37.5	18–63
Education	Incomplete/Completed Primary	59	59
	Completed Secondary	41	41
Ever injected drugs	Yes	48	48
	No	52	52
Sex in the last 6 months	Yes	95	95
	No	5	5
Migration experience			
Region of residence prior to displacement	Abkhazia	94	94
	Samachablo/Tskhinvali region (Ossetia)	6	6
Year of forced displacement	1991–1993	17	17
	2008	83	83
Type of residence immediately after relocation	Own apartment	3	3
	IDP Shelter	80	80
	Other	17	17
HIV and HCV test results			
	IDP with IDU experience	IDP without IDU Experience	Sample overall
Positive rapid HIV test (N, %)	None	None	None
Positive rapid HCV test (N, %)	11 (23%)	0	11 (11%)
Positive HCV RNA/cAg (N, %)	3 (6%)	0	3 (3%)

^*^IDP – internally displaced people

Forty-eight participants (48%) reported ever injecting drugs; and the average age at first injection was 22 years (range 14—35). Details of participants who reported IDU can be found in Table 2. The average duration of IDU experience was 6 years (range 1- 24); 8 participants (17%) started injecting drugs in the last 12 months. 52% with IDU experience (N = 25) had injected drugs in the last 30 days; the majority had never been in a Methadone/Buprenorphine + naloxone program (N = 43, 90%). Of those with recent IDU experience, 2% (N = 3) shared needles in the last 30 days.

**Table 2:** Drug use experience and sexual practices

Drug Use Experience and Sexual Practices (for PWID* only, N = 48)			
		Mean	Range
Age of First Drug Use Injection (years)		22	14–35
Duration of IDU** (years)		6	1–24

Types of Drug Injected			
		N	%
B*uprenorphine and naloxone*		23	92
Heroin		15	60
Vint or jeff (methamphetamine and methcathinone)		12	48
Participants reporting marijuana usage		24	96

		N	%
Ever been in a Methadone/Buprenorphine + naloxone program	Yes, still in the program	1	2
	Yes, but not anymore	4	8
	No	43	90
IDU in the last 30 days	Yes	25	52
	No	23	48
Shared needles in the last 30 days	Yes	3	12
	No	21	88

Sexual practices (for those who had sex in the past 6 months only, N = 95)			
		N	%
Number of male sexual partners (women)	1	26	90
	> 1	3	10
Number of female sexual partners (men)	1	25	38
	> 1	41	62
Receiving money for sex	Yes	1	94
	No	94	99
Paying money for sex	Yes	21	22
	No	74	78
Condom use when having sex	Always	35	37
	Not always	60	63

^*^PWID people who inject drugs

**IDU injection drug use

Ninety-five percent of participants reported sexual intercourse in the last 6 months; of those, 90% of women (N = 26) and 38% of men (N = 25) reported having 1 partner in that time period. No participants reported same-sex sexual partners. One woman reported sex work; 30% of men (N = 21) reported paying for sex. 37% of participants (N = 35) reported “always” using condoms when having sex in the last 6 months.

### Differences in participants with and without IDU experience

PWID were overwhelmingly male (98%) compared to non-PWID (42%; p < 0.001) and older (mean age 40 vs 35, respectively; p = 0.024). More PWID compared to non-PWID knew where to access HIV testing (98% vs 67%, p < 0.001) and HCV testing (100% vs 90%, p = 0.027), had ever been tested for HIV (94% vs 46%, p < 0.001), and HCV (96% vs 69%, p < 0.001). Primary reasons reported by non-PWID for not getting tested for HIV were “never thinking about HIV testing” (79%) and “not knowing where to take a test” (61%). Primary reasons reported by non-PWID for not getting HCV tested were similar: “never thinking of HCV testing” (75%) and “not knowing where to take a test” (31%).

All participants who reported previously testing positive for HCV (N = 6) were PWID; all reported receiving treatment and clearing infection There were no reinfections in our sample. All participants (N = 11) who tested positive with HCV in this study had IDU experience, resulting in 23% anti-HCV prevalence in IDP who inject drugs (IDPWID).

More PWID compared to non-PWID answered all questions in the HIV knowledge test correctly (35% vs 13%, p = 0.015), but there was no difference in the HCV knowledge test (70% vs 67% correct, p = 0.7).

With regards to mental health, more PWID experienced moderately severe depression compared to non-PWID (23% vs 15%, p = 0.009) and felt that their behaviours became riskier after displacement (92% vs 15%, p < 0.001). In the last year, there were no differences between PWID and non-PWID in experiences of assault from family, neighbours, roommates, or colleagues, but more PWID (compared to non-PWID) reported being assaulted by police (10% vs 0%, p = 0.022) and by other IDPs (15% vs 2%, p = 0.022).

## Discussion

Internal displacement is on the rise globally [2], making the monitoring of HIV and HCV transmission among IDPs an important task. In this exploratory work, we found evidence of a higher HCV prevalence in Georgian IDPs compared to previous reports from the general population, which can be explained by the fact that we have a high proportion of PWID in our sample. We did not find evidence of a higher HIV prevalence among IDPs with or without IDU experience compared to previously reported estimates in the general population.

While more participants in total tested anti-HCV positive (11%; all of whom had IDU experience) compared to an estimated 6.8% of the general population [11], this is likely explained by the high number of participants with IDU experience in our sample (48%). In the last general population survey conducted in 2015, an estimated 2.2% of adults reported IDU experience [27]. No reports exist on IDU prevalence among Georgian IDPs [8], but our sample likely had more PWID because recruitment was conducted by a local harm reduction NGO [8, 28]. When compared to non-displaced PWID, anti-HCV prevalence among IDPWID in this study (23%) was lower than the one reported among PWID in Gori (the regional capital of Shida Kartli) in the 2022 IBBS survey (66.7%); additionally, a simplified bio behavioural survey conducted among PWID in Gori in 2023 revealed anti-HCV prevalence of 57.7% [14]. Differences in HCV prevalence could be attributed to limited mixing between local PWID and IDPWID. Consistent with low estimates (2.3%) of HIV prevalence among PWID in Georgia, we found no HIV infections in our small sample of 48 IDPWID [12]. The Georgian HCV elimination campaign has been internationally recognized for its success; 41 specialized HCV treatment centres existed in the country as of 2018, and an estimated 72,000 people with HCV were cured between 2015 and 2020 [10, 11, 28].

The vast majority of participants knew where to get HCV tested, have been tested for HCV, and demonstrated knowledge of HCV transmission, showing evidence that previously evaluated successful HCV interventions like the 2015 country-wide HCV elimination campaign [29–31] had also resulted in higher levels of HCV knowledge among Georgians without IDU experience. At the same time, knowledge of HIV transmission and testing remains low, particularly among non-PWID. Programs aimed at increasing HIV knowledge among non-PWID can reduce stigma towards PLWH [32, 33].

In 2019, an estimated 10,500–12,000 people per month were being reached by GHRN’s harm reduction programs (about 20% of the estimated population of PWID in Georgia); PWID involved with these programs were less likely to have shared needles within 6 months and more likely to have been tested for HIV, compared to non-involved PWID [16, 34]. Despite these successes, 12% of recent PWID in our sample had shared needles and 90% had never been in a Methadone/Buprenorphine + naloxone program; furthermore, prevalence of condom use remained low. Alarmingly, a high proportion (17%) of IDPWID initiated IDU in the last 12 months despite being displaced in 2008 or earlier. High proportions of new PWID amongst IDPs create increased pools of individuals at increased risk for HIV/HCV [35] indicating a need for sustained harm reduction services. As the majority of IDPWID (92%) also reported that their behaviours became riskier after displacement, it is crucial to offer harm reduction services and conduct continuous surveillance of behaviour changes and prevalence of HIV/HCV (including testing) in internally and externally displaced PWID in Georgia, as these populations are expected to grow due to the Russian war against Ukraine [36].

While IDPs generally experience high levels of anxiety and depression [25], IDPWID participants had higher prevalence of depression than non-PWID. Correlations between substance use and depression are widely documented [37, 38]. The majority of IDPs were displaced in 2008, but the average duration of IDU experience was 6 years; thus, it seems unlikely that IDU was initiated as a coping mechanism immediately following displacement. Finally, IDPWID experience more assaults from police and other IDPs than non-PWID, likely due to widespread stigma against PWID in Georgia [34].

Intervention suggestions include developing targeted evidence-based interventions and policies using international best practices. Community-based workshops (including meetings with religious leaders) could address intracommunity stigma against IDPWID, and awareness campaigns could help dispel myths, spread factual information, and lessen stigma associated with IDU [7, 34]. Creating a stigma-free environment is a major responsibility of healthcare providers; training for healthcare practitioners about how to provide nonjudgmental, culturally competent care for PWID could help achieve this goal [39], as many PWID report experiences of stigma in medical settings as a barrier to receiving care, including HCV treatment [40, 41]. Lastly, Georgian laws against drug use are strict, and previous research points to a need for re-evaluation of national drug policy [42].

## Limitations

Cross-sectional study design makes it impossible to assess HIV or HCV incidence in our IDP sample or make causal inference with respect to various behaviours or exposures [43]. Additionally, because IDPs are a hard-to-reach population, we used a non-probability sampling technique that makes our findings less generalizable [20]. As recruitment was conducted by a NGO that provides harm reduction services, the proportion of PWID was likely higher in our sample than in the general population of Georgian IDPs. Finally, this was a pilot study, and our sample size was determined and saturated by available resources.

## Conclusions

The study revealed low HIV and HCV prevalence rates among Georgian IDPs. While IDPs have high levels of understanding of HCV, knowledge about HIV remains low. Nearly one in five IDPWID participants had initiated IDU within the past year, indicating opportunities for further research about IDU among IDPs in Georgia. As the number of displaced individuals in the region grows, preventive activities such as screenings and testing must be scaled up.

## Supplementary Information

Supplementary file 1: Table S1: (uploaded separately) HCV and HIV knowledge questions asked to participants. Supplemental Table S2: (uploaded separately) Question blocks with descriptions of the categories of questions and summaries of questions asked to participants. Table S3: (uploaded separately) Full questionnaire of questions asked to participants.

Supplementary file 2.

## Data Availability

Data available upon request from corresponding author.
