# Supplementary material for: HIV and hepatitis C Virus in internally displaced people with and without injection drug use experience in the region of Shida Kartli, Georgia
Source: BMC Res Notes. 2024 Oct 20;17:315. doi: 10.1186/s13104-024-06891-9 (PMC11492736; doi:10.1186/s13104-024-06891-9)
Supplement: Supplementary file 1 — Table S1: (uploaded separately) HCV and HIV knowledge questions asked to participants. Supplemental Table S2: (uploaded separately) Question blocks with descriptions of the categories of questions and summaries of questions asked to participants. Table S3: (uploaded separately) Full questionnaire of questions asked to participants. [file 13104_2024_6891_MOESM1_ESM.docx]

| **Supplementary Table 1: HIV and HCV Knowledge** | |
| --- | --- |
|  | |
| **HIV knowledge (participants can provide one of four answers to each question: right, wrong, I do not know, refuse to answer)** | **1. HIV infection risk decreases if you have one permanent sexual partner, who is not infected and has no sexual connection with other person**  **2. Is there a chance to reduce HIV infection risk if person uses a condom every time during sex?**  **3. Do you think that a HIV-positive person may have a healthy look?**  **4. How do you think: can a person get HIV infection by using food or water of HIV infected person?**  **5. May HIV infection be transmitted by mosquito bite?** |
| **HCV knowledge (participants can provide one of four answers to each item: yes, no, I do not know, refuse to answer)** | **Can you tell me how Hepatitis C is transmitted?**  **1. By food**  **2. By sexual transmission**  **3. Holding hands with HCV infected person**  **4. By drops (coughing or sneezing)**  **5. By sharing personal hygiene items such as razors, toothbrushes**  **6. By sharing household items such as cup, spoon, fork**  **7. By sharing used needles or syringes**  **8. Having close touch with items in public areas/spaces such as public transport and public toilets**  **9. Other (please specify)** |

| **Supplementary Table 2: Question Blocks** | | |
| --- | --- | --- |
|  | | |
|  | **Categories** | **Selected Topics** |
|  | | |
| **Question Block A** | **Socio-demographic** | **Age, date of birth, gender, location of participant, education, employment status, current residence** |
| **Question Block B** | **Migration Experience** | **Region of birth, region of residence when forced to relocate, year of forced relocation, residence immediately after relocation, duration of time in current residence** |
| **Question Block C** | **Drug Use** | **Experience with injection drug use (IDU), age at first IDU, drug use (non-injection) and IDU within 30 days, type of drug(s) used within 30 days, overdose experience within 12 months, sharing of drug use equipment within 30 days** |
| **Question Block D** | **Sexual Practices** | **Sexual orientation, number of sexual partners within 6 months, condom use within 6 months, receiving or paying money for sex within 6 months** |
| **Question Block E** | **Human Immunodeficiency Virus Knowledge and Testing and Treatment Experience** | **Level of concern about HIV, reasons for not taking HIV test, previous HIV testing experience, knowledge of HIV testing and treatment locations, anti-retroviral (ART) experience, HIV/AIDS transmission knowledge** |
| **Question Block F** | **Hepatitis C Virus Knowledge and Testing and Treatment Experience** | **Level of concern about HCV, HCV transmission knowledge, knowledge of HCV testing and treatment locations, reasons for not taking HCV test, HCV medication experience (including completion status), previous HCV treatment success** |
| **Question Block G** | **Other** | **PHQ-9 (Depression Assessment), self-assessment of risky behavior after displacement, knowledge of free HIV/HCV services, experiences of assault within 12 months** |
