# Supplementary material for: HIV and hepatitis C Virus in internally displaced people with and without injection drug use experience in the region of Shida Kartli, Georgia
Source: BMC Res Notes. 2024 Oct 20;17:315. doi: 10.1186/s13104-024-06891-9 (PMC11492736; doi:10.1186/s13104-024-06891-9)
Supplement: Supplementary file 2 [file 13104_2024_6891_MOESM2_ESM.docx]

| **A: General Information** | | | | | |
| --- | --- | --- | --- | --- | --- |
|  |  |  |  |  |  |
| A1. Today’s date |  |  |  |  |  |
|  |  |  |  |  |  |
|  |  |  |  |  |  |
|  |  |  |  |  |  |
|  |  |  |  |  |  |
|  |  |  |  |  |  |
|  |  |  |  |  |  |
|  |  |  |  |  |  |

A: General Information

A1. Today’s date.
A2. Indicate the respondent’s code.
A3. Indicate the town where the questionnaire is filled:
1. Poplars
2. Karaleti
3. Khurvaleti
4. Scra
5. Berbuk
6. Blacks
7. Sakasheti
8. Other

A4. Please indicate the respondent’s date of birth:
A5. Please indicate the respondent’s age:
A6: Please indicate the respondent’s gender (choose one):
1. Woman 2. Man 3. Non-binary

A7: Respondent’s education (choose one):
1. Incomplete secondary
2. Complete secondary
3. College or professional institution
4. Incomplete higher
5. Complete higher
99. Did not answer

A8: Current employment status (choose one):
1. Unemployed
2. Self-employed
3. Part time employed
4. Full time employed
5. Pensioner
6. Person with Disabilities
7. Student
99. Did not answer

A9. What was your personal income source for the previous month?
1. Employment
2. Any amount received by selling or renting from your own business or your properties
3. Any amount given by a friend, a relativey, a partner, borrowed money
4. Social assistance or pension
5. Illegal income
99. Did not answer

A10. How much money did you have for personal use during the last 30 days (please specify):

99. Did not answer

A11. Current residence (choose one):
1. Own apartment
2. Parents/relative/friend’s apartment
3. Rent, mortgage
4. IDP shelter
5. Wherever possible (frequent change of residence)
6. Homeless, does not have permanent shelter (includes in the street, in abandoned places, at railway stations,etc)
99. Did not answer

A12. The number of people you live with currently (in the last month)? (with the exception of minor children)

99. Did not answer

B: Migration Experience

B1: What region of Georgia were you born in?
B2: Which region of Georgia did you live in when you were forced to relocate?
B3: What year were you forced to relocate?
B4: Which region did you reside in immediately after relocation?

B5: Residence immediately after relocation?
1. Own apartment
2. Parents/relative/friend’s apartment
3. Rent, mortgage
4. IDP shelter
5. Wherever possible (Frequent change of residence)
6. Homeless, did not have permanent shelter
99. Did not answer

B6: When did you move to your current region of residence? (please indicate a year)
B7: How long have you been staying in your current residence

C: Drug Use

C1: Have you ever injected drugs?
1. Yes
2. No (Go to D1)
99. Did not answer (Go to D1)

C2: At what age did you inject first?
99. Did not answer

C3. How long did you inject drugs? (indicate in years)

C4. Have you ever been in Methadone/Suboxone program? (Indicate the current status)
1. Never
2. Now I am in the program
3. I quit last month
4. I quit just a few months ago
5. I had been in the program for more than a year ago and more
99. Did not answer

C5: Have you injected drugs in the last 30 days?
1. Yes
2. No (Go to C11)
99. Did not answer (Go to C11)

C6: During the last 30 days how many times did you use injection drugs? (Specify how many times he/she had used injection drugs)
99. Did not answer

C7: During last 30 days with how many different people did you injected drugs in one place (the maximum number)

C8. During last 30 days which drugs have you used, mark and select one by one:

| Type of usage | Injection | Per Oss | Smoke | Snorting | Have not used | Difficult to answer |
| --- | --- | --- | --- | --- | --- | --- |
| C7.1 Desomorphine (Crocodile |  |  |  |  |  |  |
| C7.2 Opioids (Tramal, Tramadol, Tetra, Morphine, Opium, “black” and others |  |  |  |  |  |  |
| C7.3 Heroin, sirets |  |  |  |  |  |  |
| C7.4 Street methadone |  |  |  |  |  |  |
| C7.5 Street suboxon/subutex |  |  |  |  |  |  |
| C7.6 Cocaine, crack |  |  |  |  |  |  |
| C7.7 Vint, Jeff |  |  |  |  |  |  |
| C7.8 Amphetamine/ methamphetamine tablets |  |  |  |  |  |  |
| C7.9 Bath salts |  |  |  |  |  |  |
| C7.10 Ecstasy, Emdema |  |  |  |  |  |  |
| C7.11 Tropicamid, ketamine, Calypso |  |  |  |  |  |  |
| C7.12 Hallucinogens (LSD, mushrooms, Psylocybin) |  |  |  |  |  |  |
| C7.13 Cannabis, Hashish |  |  |  |  |  |  |
| C7.14 Bio, bio marijuana |  |  |  |  |  |  |
| C7.15 Volatile solvents, snorting drugs |  |  |  |  |  |  |
| C7.16 Psychotropic (Xanax, benzoic, Lyrics, Grimodin, GABA-gamma, Baklosan, and etc.) |  |  |  |  |  |  |
| C7.17 Antihistamines in mixture (Dimedrol, Pipolphen, Suprastin and others) |  |  |  |  |  |  |
| C7.18 Bio-Drug |  |  |  |  |  |  |
| C7.19 Alcohol |  |  |  |  |  |  |
| C7.20 Others, verify |  |  |  |  |  |  |
|  |  |  |  |  |  |  |

C8. Did you have overdose during last year and how many times?
99. Did not answer

C9. Have you shared syringe or injection equipment with anyone during last 30 days? (If not, or have not injected, proceed)
0. No
1. I have not injected during last 6 months
2. Yes
99. Did not answer

C10. If yes, with how many different people did you share syringe during last 30 days?
0. 0
1. With 1 other person
2. With 2 or 3 other people
3. With 4 or more different people

C11. Have you had overdose during last 12 months and how many times?
99. Did not answer

D: Sexual Practices

D1. How would you describe yourself?
1. Heterosexual
2. Gay or Homosexual
3. Bisexual
4. Transgender
5. Other, please specify

Please note, for the following question, sex means any vaginal, anal, or oral intercourse
D2. With how many men have you had sex during last 6 months?
99. Did not answer
D3. With how many women have you had sex during last 6 months?
99. Did not answer

D4. How often did you take money from someone for sex during last 6 months?
0. Never
1. A few times or less
2. A few times each month
3. Once or more each week

D5. How often did you give money to someone in order to have sex with them during last 6 months?
0. Never
1. A few times or less
2. A few times each month
3. Once or more each week

D6. How often did you use condoms when you had sex during last 6 months?
0. I have not had sex in the past 6 months
1. All the time
2. Most of the time
3. Some of the time
4. None of the time

E: Human Immunodeficiency virus and testing

If you are aware about HIV positive status, skip to question E6

E1. How much are you threatened about getting HIV/AIDS?
0. Not at all
1. Slightly
2. Moderately
3. Considerably
4. Extremely

E2. Do you know where to get tested for HIV?
1. Yes
2. No
99. Did not answer

E3. Have you ever been tested for HIV before today? (please choose one option only)
1. Yes – Go to E5
2. No – Go to E4
99. Did not answer – Go to E4

E4. Have you not taken HIV test for any of these reasons? (Don’t read out, several answers are acceptable)

|  | Yes | No |
| --- | --- | --- |
| 1. I had no desire to take the test | 1 | 2 |
| 2. My sexual behavior was safe | 1 | 2 |
| 3. I have always used drugs safely | 1 | 2 |
| 4. I’m afraid of understanding my HIV status | 1 | 2 |
| 5. I think testing is expensive | 1 | 2 |
| 6. I did not have time for that | 1 | 2 |
| 7. Testing locations are inconvenient | 1 | 2 |
| 8. I do not know where I can get tested for HIV | 1 | 2 |
| 9. I do not want anyone else to know my status /  I am afraid | 1 | 2 |
| 10. I never thought about HIV testing | 1 | 2 |
| 11. Other (please specify) | 1 | 2 |
| 99. Did not answer | | |

‘

E5. Did you know the result of your HIV testing? (choose one):
1. Never had HIV testing – Go to E8
2. I did not receive answer – Go to E8
3. I am HIV negative – Go to E8
4. I am HIV positive – Go to E6
99. Did not answer – Go to E8

E6: Are you on the antiretroviral therapy (ART)? (please choose one option only)
1. Yes
2. No, but I have already been prescribed ART – Go to E8
3. I used to take it, but I have stopped – Go to E7
4. No – Go to E8
99. Did not answer – Go to E8

E7. Sometimes it is challenging for people to take HIV drugs (ART) because of personal situations or circumstances. Why have you stopped or not yet started taking ART? (Don’t read out, several answers are acceptable)

| Statements | Agree | Disagree |
| --- | --- | --- |
| 1. I think I do not need it | 1 | 2 |
| 2. I have no time to go to hospital | 1 | 2 |
| 3. I have no energy or motivation to go to hospital | 1 | 2 |
| 4. I have skipped a visit, so it is hard to go back | 1 | 2 |
| 5. I was too sick to go to hospital | 1 | 2 |
| 6. My drug use prevented my from going to hospital | 1 | 2 |
| 7. I had adverse effects or complications | 1 | 2 |
| 8. I could not afford the pills, it was too expensive | 1 | 2 |
| 9. I do not understand when I need to take each of the pills | 1 | 2 |
| 10. I sold/lost the drugs, or they were stolen | 1 | 2 |
| 11. My alcohol use prevented me from going to hospital | 1 | 2 |
| 12. I had to wait for too long, and that is why I just left unnoticed | 1 | 2 |
| 13. I’m in a waiting list | 1 | 2 |
| 14. The AIDS Centre is too remote + I have no money to travel there | 1 | 2 |
| 15. Stigma from staff | 1 | 2 |
| 16. Stigma because of HIV | 1 | 2 |
| 17. Someone would find out that I inject drugs | 1 | 2 |
| 18. Possible or experienced violence | 1 | 2 |
| 11. Possible or experienced prosecution or detention by law enforcement officers | 1 | 2 |

Knowledge about HIV/AIDS

E8. For each question mark one correct answer

|  | Right | Wrong | I do not know | Refused to answer |
| --- | --- | --- | --- | --- |
| 1. HIV infection risk decreases if you have one permanent sexual partner, who is not infected and has no sexual connection with other person | 1 | 2 | 88 | 99 |
| 2. Is there a chance to reduce HIV infection risk if person uses a condom every time during sex? | 1 | 2 | 88 | 99 |
| 3. Do you think that a HIV-positive person may have a healthy look? | 1 | 2 | 88 | 99 |
| 4. How do you think: can a person get HIV infection by using food or water of HIV infected person? | 1 | 2 | 88 | 99 |
| 5. May HIV infection be transmitted by mosquito bite? | 1 | 2 | 88 | 99 |

F: Hepatitis C Virus

F1. How much are you threatened about getting Hepatitis C Virus?
0. Not at all
1. Slightly
2. Moderately
3. Considerably
4. Extremely
99. Did not answer

F2. Could you tell me how is Hepatitis C transmitted? (read the question to the respondent, circle all possible answers)
1. By food
2. By sexual transmission
3. Holding hands with HCV positive person
4. By droplets (coughing, sneezing)
5. By sharing personal hygiene items, such as: razors, toothbrushes
6. By sharing household items, such as: cup, spoon, fork
7. By sharing used needle or syringes
8. By having close touch with items in public areas/places such as public transport and public toilets
9. Other (please specify)

F3. Do you know where to get tested for Hepatitis C?
1. Yes
2. No
99. Did not answer

F4. Have you ever been tested for HCV (Mark the latest testing)
1. Yes, during the last 2 years – Go to F6
2. Yes, from 2 to 5 year period – Go to F6
3. Yes, 5 years ago – Go to F6
4. No – Go to F5
5. Don’t know – Go to F5
99. Did not answer

F5. Please indicate the reason why you didn’t take an HCV test (Don’t read out, several answers are acceptable)

|  | Yes | No |
| --- | --- | --- |
| 1. I had no desire to take the test | 1 | 2 |
| 2. My sexual behavior was safe | 1 | 2 |
| 3. I have always used drugs safely | 1 | 2 |
| 4. I’m afraid of understanding my HCV status | 1 | 2 |
| 5. I think testing is expensive | 1 | 2 |
| 6. I did not have time for that | 1 | 2 |
| 7. Testing locations are inconvenient | 1 | 2 |
| 8. I do not know where I can get tested for HCV | 1 | 2 |
| 9. I do not want anyone else to know my status /  I am afraid | 1 | 2 |
| 10. I never thought about HCV testing | 1 | 2 |
| 11. Other (please specify) |  |  |
| 99. Did not answer |  |  |

F6: Have you ever taken medications to treat your Hepatitis C infection?
1. Yes, during the last 2 years
2. Yes, from 2 to 5 year period
3. Yes, from 5 years ago
4. No
88. Don’t know
99. No response

F7. Why didn’t you take medication for your Hepatitis C infection? (Don’t read out, several answers are acceptable). Then go to Section G.

|  | Yes | No | Don’t know / don’t remember |
| --- | --- | --- | --- |
| 1. I’m not infected and do not need treatment | 1 | 2 | 88 |
| 2. I do not have time to go to the hospital | 1 | 2 | 88 |
| 3. I have no energy or motivation to go to the hospital | 1 | 2 | 88 |
| 4. I missed the visit so it is hard to come back | 1 | 2 | 88 |
| 5. I was so ill I could not go | 1 | 2 | 88 |
| 6. Drug use prevented me | 1 | 2 | 88 |
| 7. Due to side effects and possible complications | 1 | 2 | 88 |
| 8. The medicine was very expensive, not available | 1 | 2 | 88 |
| 9. I do not understand when I should take medication | 1 | 2 | 88 |
| 10. I sold/lost drugs, or they were stolen | 1 | 2 | 88 |
| 11. Alcohol consumption prevented me | 1 | 2 | 88 |
| 12. I had to wait too long and so I left | 1 | 2 | 88 |
| 13. I am on the waiting list for treatment | 1 | 2 | 88 |
| 14. The medical centre is very far away + I do not have the money to travel there | 1 | 2 | 88 |
| 15. Stigma from medical staff | 1 | 2 | 88 |
| 16. Self-stigma because of HCV | 1 | 2 | 88 |
| 17. Threatened that people know I use drugs | 1 | 2 | 88 |
| 18. Possible or experienced violence | 1 | 2 | 88 |
| 19. Possible or experienced prosecution or arrest by law enforcement | 1 | 2 | 88 |

F8. Did you complete your Hepatitis C treatment, or did you stop before the end?
1. I completed the treatment.
2. I stopped treatment before the end.
3. Currently taking a treatment.
88. Don’t know
99. No response

F9. What was the reason for stopping the treatment before the end?
1. Side effects of treatment
2. Treatment provider was geographically far away
3. Medical institution / doctor’s negative attitude / stigma and discrimination
4. Other (please specify)
88. Don’t know
99. No response

F10. Did the treatment cure your Hepatitis C infection? (A cure is sometimes referred to as “sustained virologic response.”)
1. Yes
2. No.

88. Don’t know
99. No response

G. Other

G1. Now I will ask you a question about how you feel, your emotions and thoughts, about depression. Have you encountered any of the following problems in the last two weeks? (please choose one option in each line).

| Statement | Nothing like that | Yes, 1 or 2 days | Yes, 3 or 4 days | Yes, almost every day (5-7 days) |
| --- | --- | --- | --- | --- |
| 1. Reduction of interest or satisfaction of completed tasks | 0 | 1 | 2 | 3 |
| 2. Bad mood, feeling dispirited or hopeless | 0 | 1 | 2 | 3 |
| 3. Problems with falling asleep, superficial sleep, or on the contrary, excessive drowsiness | 0 | 1 | 2 | 3 |
| 4. Feeling tired and feeble | 0 | 1 | 2 | 3 |
| 5. Bad appetite or overeating | 0 | 1 | 2 | 3 |
| 6. Negative feeling about self – feeling like a loser, a person who let himself/herself or his/her family down | 0 | 1 | 2 | 3 |
| 7. Problems with focusing, e.g. while reading a newspaper or watching TV | 0 | 1 | 2 | 3 |
| 8. Slow movement or speech notable even for people around you. Or, on the contrary, excessive activity and restlessness uncharacteristic for you | 0 | 1 | 2 | 3 |
| 9. Thinking it would be better if you died, thoughts of self-harm | 0 | 1 | 2 | 3 |

G2. Do you think your risky behavior has changed as a whole (injecting drug use, excessive alcohol use, unprotected sex) after becoming an internally displaced person?
1. Yes
2. No
88. Don’t know
99. No response

G3. Have you heard of free HIV testing centers or mobile ambulatories?
1. Yes
2. No
88. Don’t know
99. No response

G4. Have you ever heard of free HCV testing centers or mobile ambulatories?
1. Yes
2. No
88. Don’t know
99. No response

G5. Have you ever heard about HIV prevention services where syringes, needles, and condoms are distributed free of charge?
1. Yes
2. No
88. Don’t know
99. No response

G6. If you’ve heard and know about them, have you received their services?
1. Yes I have personally visited and received the service/injection equipment
2. HIV and HCV tests in mobile ambulatories
3. Yes I have heard and received syringes, condoms, newsletters, or other materials from my friend
4. No, I have not heard, for the first time I hear about such programs
88. Don’t know
99. No response

G7. Have you ever heard about Naloxone?
1. Yes
2. No
88. Don’t know
99. No response

G8. If yes, where have you gotten Naloxone?
1. From harm reduction program
2. From a friend
3. From pharmacy
88. Don’t know
99. No response

G9. What kind of service do you think would be available for free?
1. Testing for HIV
2. Testing for Hepatitis C
3. Consult a doctor
4. Consult a psychology
5. Consult a lawyer
6. Syringes and needles
7. Naloxone
8. Condoms
9. Trainings, risk counseling
10. Peer training, risk counseling
11. Drugs for vein care
12. Information materials
13. HCV RNA test

G10. Have you been tested for COVID in the last 12 months and if so, what was the test result?
1. Yes, once, positive
2. Yes, more than once, positive at least once
3. Yes, negative
4. Haven’t had a test
99. Don’t remember/refuse to answer

G11. Was there any assault on you (physically or verbally) in the last 12 months?

|  | Yes | No | Don’t remember/don’t know | No answer |
| --- | --- | --- | --- | --- |
| 1. Your family members | 1 | 2 | 88 | 99 |
| 2. Your neighbors/roommates | 1 | 2 | 88 | 99 |
| 3. Your colleagues | 1 | 2 | 88 | 99 |
| 4. Police | 1 | 2 | 88 | 99 |
| 5. People who inject drugs | 1 | 2 | 88 | 99 |
| 6. Men who have sex with men | 1 | 2 | 88 | 99 |
| 7. Sex workers | 1 | 2 | 88 | 99 |
| 8. Transgender people | 1 | 2 | 88 | 99 |
| 9. IDPs | 1 | 2 | 88 | 99 |
| 10. Other (please specify) | 1 | 2 | 88 | 99 |

G12. Which devices do you use to access the Internet? (choose all that apply)
1. Desktop at home
2. Desktop at friend’s/relative’s place
3. Mobile phone
4. Smartphone
5. Laptop
6. Tablet
7. Other (specify)
88. Don’t remember / don’t know
99. No answer (don’t read out loud)

G13. In case a person does not take coupons for recruitment, please collect reasons in the end of the interview
1. I am not sure I will find anyone interested in study
2. I don’t have desire / I am lazy in such issues
3. Other (please specify)

G14. As a participant whether he/she would decide to participate in next study if they also had to donate 5mL of blood for further testing
1. I will participate
2. I will not participate
88. I don’t know
99. No answer
